# Communication in mental health nursing - Bachelor Students' appraisal of a blended learning training programme - an exploratory study

**DOI:** 10.1186/s12912-018-0288-9

**Published:** 2018-05-15

**Authors:** Merete Furnes, Kari Sofie Kvaal, Sevald Høye

**Affiliations:** 1 Faculty of Health and Social Services, Inland Norway University of Applied Sciences, Elverum, Norway; 2 Faculty of Nursing and Health Sciences, Nord University, Bodø, Norway

**Keywords:** Blended learning, Role play, Communication skills, Mental health nursing, Video, Feed-back

## Abstract

**Background:**

It is important that mental health nursing students at Bachelor level obtain effective communication skills. Many students dread the fact that in the mental health field they will encounter patients and relatives with various backgrounds and personalities. Large classes and limited teaching resources in nursing education are challenging. To prepare students for mental health nursing practice, a communication skills course based on the blended learning method was developed and carried out at two different campuses.

The aim of the study is to explore Bachelor nursing students’ appraisal of blended learning methods for enhancing communication skills in mental health nursing.

**Methods:**

This study employed an exploratory design. Teaching and information materials were available on the learning management system (LMS). Videotaped role play training was carried out in the Simulation Department. Data were collected after the course by means of a questionnaire with closed and open-ended questions. The response rate was 59.2%. Quantitative data were analysed using the Statistical package for the Social Sciences (SPSS) and the Kruskal Wallis test, while qualitative data were analysed by content analysis based on Graneheim and Lundman’s approach.

**Results:**

No impact of background variables was observed. Students appreciated teachers’ participation in role play and immediate feedback was considered especially important for learning outcomes. The students perceived that their communication skills and knowledge had improved after completing the blended learning programme.

**Conclusions:**

According to the nursing students, blended learning is an appropriate method for improving communication skills in preparation for mental health nursing. Blended learning makes it possible to build flexible courses with limited resources.

**Electronic supplementary material:**

The online version of this article (10.1186/s12912-018-0288-9) contains supplementary material, which is available to authorized users.

## Background

Effective communication is a fundamental element in all nursing and forms an integral part of quality patient care [1, 2]. The National Curriculum for Nursing Education in Norway sets out requirements related to nurses’ communication skills after the three-year bachelor degree programme. Nurses must be able to encounter patients and their families with sensitivity, empathy and moral accountability. They should also have the ability to communicate with people of different ethnic, religious and cultural backgrounds who have a range of personalities, as well as being able to teach and guide patients, families, staff and students [3].

Nursing students with a native language other than that of the majority of their peers, might face an additional challenge when it comes to communication skills. Language competence is described as a prerequisite for good communication skills [4].

Internationally, good communication skills are described as an important part of nurses’ core competencies, crucial for nursing practice and patient-centred care [5]. The way patients are greeted by nurses and other health professionals will affect how health care is provided [6, 7].

In addition, communication is important for patient safety. The Joint Commission on Accreditation of Healthcare Organizations (JCAHO) claims that 65% of adverse events or incorrect treatment is associated with communication failure [8]. In its description of core competencies in the health profession, the US Institute of Medicine recommends increased focus on improving professional communication, cooperation and a patient-centred approach in order to strengthen patient safety [8, 9].

Nursing students encounter patients with various issues during the Bachelor Programme. During mental health nursing training, students encounter patients with dementia, anxiety, depression and other serious mental disorders. Students often do not look forward to mental health nursing practice. The field is unfamiliar to most students and the communication demands are large [10]. Many people, including nurses, have prejudices against psychiatric patients [11]. Stevens et al. (2013) describe students’ dread of psychiatric patients as a stereotypical fear of people with mental illness [12]. In the interpersonal relationship between nurses and patients or their relatives, nurses encounter diverse emotions, which is also the case in mental health care. There are no difficult patients, but challenging relationships. In order to communicate effectively, nurses must acknowledge their own emotional responses [13]. Studies demonstrate that the way nurses encounter patients influences the patients’ level of aggression [14]. Treating patients in an appropriate manner requires not only self-awareness on the part of nurses, but also an understanding of the specific behaviours they may find challenging [15].

Simulation of communication in nursing practice provides an opportunity to prepare for practice before encountering real patients [16–18]. Role play is a core component of the development of processing skills. The design of role play can vary. Standardized Patients (SPs), often actors trained to simulate specific illnesses or conditions, might be used. Another possibility is peer role play, a method that is easier and cheaper to implement. Both methods seem to be valuable for communication skills training [19]. However, simulation has little tradition and a short history within mental health nursing. Studies have revealed that teaching methods vary widely between different countries, academic institutions and clinical settings [20, 21]. Some studies recommend integrated therapeutic communication training in mental health care during the nursing education to increase students’ confidence in their communication skills. However, it has also been stated that more research on this topic is required [21–23].

Simulation as a learning tool should be combined with other established principles of learning, such as group work and lectures. The face-to-face lecture tradition can make students passive listeners. In Norwegian Nursing Education, half of the bachelor programme takes the form of clinical training in hospitals or community health care. At Inland Norway University of Applied Sciences (INN), more resources are used for supervision in the clinical part of the programme than in the theoretical part. As higher education lectures in Norway are not mandatory, attendance at such lectures has decreased and students have called for a variety of methods. In Report no.16 (2016–2017), the Norwegian Government provides guidelines for more active and varied learning methods in higher education [24]. The use of blended learning has increased in combination with technological advancement [25]. Blended learning is a relatively new pedagogical framework that includes several methods [26]. One such method is the so-called «flipped classroom», where the time usually devoted to lectures is used in other ways. Lectures can be recorded on video and made available to students on the Learning Management System (LMS), after which the teachers meet the students for discussion, guidance and reflection on the content of the recorded lectures [27]. The advantage of asynchronous video recorded lectures is that the student can play them at any time, take breaks and watch them on several occasions [27, 28].

There is a demand for low-cost and effective methods in nursing education that lead to the desired learning outcomes. The intention of this study is to contribute an experience of blended learning to meet this demand. Students’ experience of this method is described in postgraduate programmes [29]. However, few studies have explored the experience of simulation in mental health nursing as a part of the blended learning methodological framework at bachelor level [30]. In the present study we wish to investigate which background variables influence the outcome of the method.

The aim of the study is to explore Bachelor nursing students’ appraisal of blended learning methods for enhancing communication skills in mental health nursing.

The research questions were:

1. Are gender and native language associated with nursing students’ appraisal of learning outcomes?
2. In what way does the presence of teachers influence students’ learning outcomes regarding therapeutic communication?
3. How do students appraise web-based lessons, videotaped role play and reflectiongroups in terms of learning outcomes?

## Methods

### Design

An exploratory design was used and a questionnaire developed to gather both quantitative and qualitative data.

### Participants and setting

This study is based on students’ assessments of a communication skills course with focus on mental health nursing and psychiatry at bachelor level. The course was part of the third year of the bachelor programme, thus the participants had over 2 years of experience as nursing students. The communication skills course took place during the first 3 weeks of a 20 week mental health nursing programme, including practical training at several mental health institutions. Immediately after the course, the students went to various institutions for mental health nursing practice. The nursing department at INN is divided into two classes on two different campuses, one large and one small, located in cities 100 km apart from each other. All lectures are common to both classes and are conducted by lecturers in an auditorium at the large campus. The lectures are video-taped and transmitted synchronously to the small campus, where the students watch the lecturer on a large screen.

The communication skills course was conducted simultaneously in the two campuses. The large campus, which is the main campus, has 165 nursing students in each class. The majority of teachers and the university administration are located at this campus. The small campus has 40 students in each class with only a small number of teachers. 75% of the students are aged between 21 and 30 years, and almost 20% have a native language other than Norwegian.

### Learning outcome

The main learning outcome of this course was the enhancement of students’ communication skills in mental health nursing. Learning outcomes were based on the European Qualification Framework (EQF) [31] . The expected learning outcomes were knowledge of basic therapeutic communication terms, knowledge of and skills in verbal and non-verbal communication between nurse and patient, and knowledge of and skills in affective awareness and tolerance when nurses encounter difficult and challenging emotions from patients and relatives. The expected learning outcomes related to competence were ability to connect theory and practice and to reflect on one’s own communication in various situations.

### Intervention

As there are over 40 different courses at the largest campus, and only three auditoriums in which video transfer is possible, synchronous lectures for the students presents great challenges. To solve this dilemma, a digital presentation of asynchronous content was developed, to ensure that nursing students could prepare themselves for clinical training in mental health nursing.

The programme consisted of two main areas:

- E-learning materials on the LMS, which were provided 2 weeks in advance to enable the students to prepare for the intervention (weeks one and two).
- Simulation by role play. The role play was videotaped and formed the basis for the subsequent reflection group comprising the teacher and students (week three).

The LMS E-learning materials consisted of information about the objectives, learning outcomes and time schedules. In addition, videos with lectures on communication theory and techniques, examples of communication situations and detailed descriptions of the patient cases were also available. The lectures emphasised the learning outcomes. No lectures were given in the traditional auditorium face-to-face form.

Communication training was performed as role play carried out in the Simulation Department. Four patient case “stations” were established. Each station represented one simulation situation: anxiety and depression, psychosis, dementia and relatives. The students were divided into groups of four. In each group, four roles were described. At the small campus the roles were patient, nurse, video photographer and observer. Teachers were not present during the role play at the small campus. At the large campus the roles were nurse, video photographer and observers, while the patient role was played by teachers with experience of mental health nursing. The roles changed for each patient case station, so that all students experienced different perspectives and everyone could play the nurse role. The students selected the situation in which they would play the role of nurse in advance, giving them the opportunity to prepare for the patient case they would encounter.

Every role play situation contained communicative and emotional challenges. All situations were videotaped by one of the group with a camera on a tablet or mobile device. Immediately after the role play, the person who played the patient gave feedback on how she/he experienced the communication with the nurse. The day after the simulation, each student group met a teacher for reflection at both the small and the large campus. This teacher had not participated in the role play at the large campus the previous day. Due to limited time, each group was only allowed to choose one video recording of the nurse-patient situation for reflection. Students and teacher viewed the video together, and focused on communication skills; what was positive and what could be improved.

### Measurement

The questionnaire was developed, adapted and tailored to measure the extent to which the students perceived that they had achieved the learning outcomes, which is in line with the European Qualification Framework [31]. In addition, the questionnaire was designed to measure the students’ assessment of the blended learning methods employed during the communication course. The face validity was critically appraised by means of discussions in a reference group, after which the authors revised the questionnaire based on consensus among the reference group members. The reference group consisted of both clinical nurses and researchers including a Professor in Mental Health Clinical Nursing and an Associate Professor in Clinical Nursing at Inland Norway University of Applied Sciences (INN University). The questionnaire was written in Norwegian, but has been translated into English by an Assistant Professor whose first language is English.

The questionnaire has seven overall themes comprising various items. The ordinal items were divided into five levels from 1 (not at all) to 5 (to a high degree). Two of the themes were formulated as statements, with five levels from 1 (totally disagree) to 5 (totally agree).

For background information, we asked for the students’ age (grouped into < 20 years, 21 to 29 years and 30>), gender (dichotomized as male/female), health care work experience before starting nursing education (dichotomized as yes/no) and native language (dichotomized as Norwegian/foreign).

In addition, we used three open-ended questions; (i) Which patient case was perceived as the most useful and why? (ii) What element of the programme led to the greatest learning outcome? and (iii) Have you any suggestions or recommendations for changes to the course?

### Data collection

At the conclusion of the training programme the students individually filled in a paper version of the questionnaire, which was distributed by teachers from the Institute of Nursing Sciences, INN University. Some of the students started their clinical practice immediately after the course and for that reason received the questionnaire with a stamped addressed envelope to facilitate its return to the researchers.

### Data analysis

The analyses were performed using the SPSS (Statistical Package for the Social Sciences), version 23. The answers were entered into the statistical program by one of the authors (MF).

The Kruskal Wallis test was employed for comparison.

The answers to the open- ended questions were analysed by content analysis based on Graneheim and Lundman [32]. The responses were transcribed verbatim and transferred into meaning units and condensed meaning units. In addition, short comments were taken into account and categorized.

### Ethical considerations

Approval was obtained from the head of the Department of Nursing at INN University, due to the desire for continuous improvement of teaching methods.

The students received oral information about the assessment of the training programme before the questionnaires were distributed. Written information was enclosed with the questionnaire. The students were guaranteed complete anonymity and informed that participation was voluntary. Furthermore, the written information contained details about the purpose of the study. The participants gave their consent by filling out the questionnaire.

The students completed the questionnaire anonymously and it was not possible to link the responses to any individual.

Approval was granted by the Norwegian Centre for Research Data (NSD). NSD Reference Number 35719. The NSD provides protection for both data and human subjects.

## Results

In total, 169 questionnaires were distributed to the students and 100 were returned (response rate 59.2%), of which 88% were from women. A total of 12% of the students had a native language other than Norwegian. 69% had health care work experience before they started the nursing education. Both the small and the large campus were represented in the respondent group, 14 and 86% respectively.

Neither gender, native language nor health care work experience had significant impact on students’ assessment of the course.

### Assessment of learning outcomes

The results revealed that the students’ communication knowledge and skills increased. They perceived that they had become familiar with the key Assisting Communication terms to a large and very large extent (73%). A similar number (71%) became familiar with the various elements of Active listening to a large and very large extent. Even more (90%) indicated that to a large and very large extent they had become familiar with and gained an understanding of how important it is for nurses to be emotionally aware and tolerant when meeting patients and relatives. In addition, 78% expressed that to a large and very large extent they could combine theoretical and practical knowledge when caring for patients suffering from psychological problems and their relatives. Almost all participants (94%) indicated that to a large and very large extent they reflected on their own communication methods when meeting patients, relatives and fellow students/colleagues.

### Assessment of the importance of blended methods

The digital materials presented on the LMS were considered important to a large and very large extent by 55%, while 41% indicated that the importance was medium and 4% that they were of some importance.

The use of role play was considered to be of large and very large importance by 62%. Only 12% evaluated it as important to some extent or not at all. Assessment of role play combined with videotaping was deemed important to a large and very large extent by 57%, while 13% indicated that it was of some or no importance. The combination of training through videotaped role play and reflection on the video the following day was considered important to a large and very large extent by 62% of the students, while 15% considered it of some or no importance.

In response to the question about which simulated cases led to the best learning outcomes, it was found that psychosis had the highest score (65%), followed by anxiety and depression (17%), care givers’ role (11%) and dementia (7%) (Table 1).

**Table 1:** Comparison of students’ assessment of methods and learning outcome when teachers were present and absent by means (95% CI) using the Kruskal Wallis test

1a) Assessment of the use of role play and video for communication skills training	Teachers present	Teachers absent	*p*-value
I learned more about how I can communicate by using role play than I would without using role play	3.9 (3.7 to 4.2)	3.1 (2.6 to 3.7)	*P* < 0.01
I learned more about my own communication skills through seeing myself on video than I would have done without using video	3.7 (3.5 to 4.0)	2.9 (2.2 to 3.5)	*P* < 0.01
1b) Assessment of learning outcomes and goals	Teachers present	Teachers absent	*p*-value
I am familiar with the key Assisting Communication terms	3.8 (3.7 to 3.9)	4.0 (3.6 to 4.4)	0.24
I am familiar with the various elements of Active listening	3.9 (3.7 to 4.0)	3.8 (3.7 to 3.9)	0.66
I am familiar with and have an understanding of how important it is for nurses to be emotionally aware and emotionally tolerant when meeting patients and relatives	4.2 (4.1 to 4.4)	4.3 (3.9 to 4.6)	0.80
I can combine theoretical and practical knowledge when meeting patients suffering from psychological problems and their relatives	4.0 (3.8 to 4.1)	3.9 (3.5 to 4.2)	0.52
I reflect on my own communication methods when meeting patients, relatives and fellow students/colleagues	4.4 (4.3 to 4.6)	4.2 (3.7 to 4.6)	0.13
1c) Assessment of the importance of methods used in the teaching and practice programme.	Teachers present	Teachers absent	*p*-value
Importance of training through role play	3.8 (3.6 to 4.0)	3.4 (2.9 to 3.9)	0.12
Importance of training through role play combined with video	3.6 (3.4 to 3.9)	3.4 (2.8 to 4.0)	0.27
Importance of training through role play, video and feedback	3.8 (3.5 to 4.0)	3.2 (2.6 to 3.9)	0.07

The presence and absence of teachers only had a significant difference on the students’ assessment of learning communication skills by means of role play and video (*p* < 0.01).

### Qualitative approach

The students were asked to elaborate on their answers to the following three open-ended questions: Which of the patient cases provided the greatest learning benefit? (Table 2); Which part of the programme provided the best learning outcomes? (Table 3); and Have you any suggestions for future communication courses?

**Table 2:** Learning outcomes of patient cases

Themes	Well prepared to meet the patients							
Categories	Increased knowledge about the patient			Teachers’ participation is important			Learning communication through role play	
Sub categories	Little previous experience of mental health	Challenging situation	Relevant situations	Feedback on communication	Sharing experiences	Simulated situations must be realistic	The nurse role was challenging	Have to be prepared when playing the nurse

**Table 3:** Overall learning outcomes

Themes	Ready for practical studies					
Categories	Increased self-awareness			Increased knowledge awareness		
Sub categories	More confident in own communication	Awareness of own strengths and weaknesses	Techniques adapted to situations	Syllabus increased communication knowledge	Learning customized subject material in LMS	Examples increased understanding of mental health

Abbreviations: *LMS* Learning Management System

When the students explained the reasons for their choice of the patient case that benefitted them the most, they appeared to emphasise the value of being prepared to encounter patients in mental health care practice. Three categories with subcategories emerged (Table 2):

### Increased knowledge about the patient

Many students highlighted the importance of realistic situations for giving them an opportunity to encounter patients and situations of which they had little previous experience.

“*The case involving psychosis was the most unfamiliar to me and therefore an informative and useful situation that made me better prepared for the upcoming psychiatric practice*”. *“Had not seen psychotic patients previously. Learned a lot by meeting such a patient”* (‘Little previous experience of mental health’).

“*But I think the next of kin provided a good learning outcome as I find conversations with relatives “scary”!”* (‘Challenging situations’).

“*It was a new experience to see these situations in real life and the actor was very good at playing his role*” (‘Realistic situation’).

### Teacher participation is important

Students have more confidence in teachers’ knowledge about the mentally ill patient than that of their fellow students. This is important for feedback, for sharing their own experiences and for making the role play realistic.

The following statements exemplify the subcategories:

“*Discovered my challenges and was inspired to read more”. “Got constructive feedback on what the nurse could have done better”* (Feedback on communication).

*“Learned how to calm the patient and divert her/his attention”. “The teacher told me about her own encounter with psychotic patients”* (Sharing experiences).

*“VERY good that teachers played the patient!” “Great involvement on the part of the teacher, which motivated me to read more”*. “*Felt that the day did not live up to my expectations as we were left to ourselves and the role play performed with fellow students was guesswork and false”* (Simulated situations must be realistic).

### Learning communication through role play

Most of the answers indicate that role play is effective for learning and developing communication skills. Playing the nurse is demanding, but educational. The subcategories are exemplified by the following statements:

*“It was an ordeal taking care of the relatives and making them feel comfortable”* (The nurse role was challenging).

“*It was this patient situation I played as a nurse, so I had to gain knowledge of the diagnosis and communication in advance*” (Have to be prepared when playing the nurse).

The most important outcome after completion of the course seems to be that the students had increased their communication and mental health nursing competence and were ready for practical studies (Table 3).

Two categories are underpinned by subcategories and exemplified by the following statements:

### Increased self-awareness

“*Role play with filming made me reflect on what happened during the meeting with the patient, become more confident about the fact that what I did that worked and enabled me to see what needs to be adjusted in relation to appropriate communication”* (More confidence in own communication).

*“Seeing myself on film was very informative, because I was able to observe my strengths and choices, which were rather bad. I could never have gained so much awareness of this had I not been able to see myself”* (Awareness of own strengths and weakness).

*“The main thing I got out of this was seeing how we resolve situations in different ways and can discuss and make new observations the next time*.” “*I learned about emotional consciousness and tolerance, transference and countertransference, body language and awareness”* (Techniques adapted to situations).

### Increased knowledge awareness

*“Read a lot more on theory and got a better overview of the basic concepts of communication and linked theory to practice experience.” “Read the syllabus, then I could use that experience to improve my communication skills”* (Syllabus increases communication knowledge).

*“Teaching videos on Fronter were very elaborate and emphasised the need to be prepared for the day in the Simulation Department”* (Learning customized subject material in the LMS).

### Recommendations for improvement

Twenty-five students provided short comments to the last open question: ‘Any suggestions for improvements?’ The students had some suggestions for how to improve the course, although several pointed out the parts that are worth continuing.

*“We could have had even more role play with filming. I think I would learn more with simulation of additional cases.”*

*“Continue to have teachers as “patients”. It was more real, without any nonsense! A teacher or otherwise a professional actor due to their greater experience of what a person with mental illness is like, makes it more realistic”* (Large campus).

*“Continue the role play, but filming leaves one very nervous and makes it difficult to learn!”*

Some students mentioned the wish for feedback: “*Standardized, immediate feedback to ensure the same feedback to all students. It is important that all teachers who play the patient provide feedback on what could be improved. Also important to point out what was good.”*

## Discussion

This study aimed to explore Bachelor nursing students’ appraisal of an educational course based on blended learning in order to enhance communication skills in mental health nursing.

The main finding is that the students were satisfied with the overall programme. They perceived that their communication skills and knowledge had improved after completing the blended teaching methods.

The result was little affected by the students’ background. One might expect that a native language other than Norwegian would influence and perhaps have a negative effect on the perception of the communication learning outcome [4], but this was not the case in the present study. Other studies demonstrate that men have a more positive attitude towards digital tools than is the case with women [33, 34], while another study indicates little difference in gender attitudes [35]. The present study reveals no significant gender differences.

Teachers have a significant impact on the students’ perception of increased communication skills. Students at the small campus who played the patient role themselves rated the learning outcomes of role play and filming lower than the students at the large campus. Thanks to their experience of nursing patients with mental disorders and their families, the teachers at the large campus who played the role of the patient and relative created an impression that the simulation was realistic. The students were confident that the teachers’ performance of the patient and relative roles was in line with reality.

Encountering standardized patients in a safe simulation situation is a way to imitate clinical settings and can make students more prepared to meet real patients. The study shows that the situations must be considered realistic, which may explain the high score on the importance of teachers being present in the Simulation Department. Few studies have compared these two methods [19], but a previous study shows that students perceive that both alternatives are valuable preparation for practice [36]. However, the same study concludes that the use of standardized patients is rated higher by nurse supervisors. The students at the small campus were well aware that teachers played the patient role at the large campus. This might have influenced their motivation and attitude to the course, and could be a reason for the great difference in perceived learning outcome.

Immediate feedback was considered more important than the reflection group the following day. Those who played the “patient” gave immediate feedback after completing the role play, which was highlighted as valuable. Students found that the feedback was provided differently, depending on the teacher. The content of the feedback was not planned in advance. As a result, the quality of the feedback from the “patient” to the student who played the nurse was quite unsystematic. The study shows that standardized feedback is critical for optimal learning. An instruction manual about what feedback should contain was found to be important in other studies [8, 37, 38]. which may indicate that it is not the teacher’s presence that is essential, but that students encounter a standardized patient. However, the present study cannot confirm this supposition.

Some statements reveal that students made new discoveries by watching themselves on video, which is confirmed by other studies [39]. Students rated role play using video somewhat lower than role play without video. This can be understood in several ways: All the students in the group want to be seen, which is an elementary, basic need. The reflection group did not reflect on all film scenarios, only one of four chosen by the group. Another explanation is that reflection on one’s own communication on video is difficult, because the reflection process is removed from introspective explanations [40, 41]. Seeing each individual student in a class of 100–200 is a challenge for educators. Therefore, learning situations like those in the present study should be organized in a way that ensures feedback to each student and an opportunity to reflect on everyone’s communication. Other studies pinpoint the same problem and suggest increased use of peer learning to resolve this challenge [42].

Students perceived the blended learning programme as an important preparation for practice. Several responses indicate that students had some negative expectations of mental health nursing practice. It is considered especially challenging for nursing students to cope with difficult emotions. Changing attitudes to mental health nursing practice is not explicitly measured in this study, but previous studies show that experience of working with psychiatric patients has a positive impact on health workers’ attitudes [43]. A large proportion responded that their knowledge of the importance of emotional awareness and tolerance when meeting patients and their relatives increased by a large or very large extent as a result of the teaching programme. This corresponded with the percentage stating that the most unfamiliar patient situations were those that provided the greatest opportunity for learning. They learned the most from especially challenging situations, such as meeting a patient with psychosis. The situations in which the students encountered a patient with dementia were ranked as the least interesting (6%). This can provide a broad picture of the students’ expectations of mental health practice. Having met patients with dementia earlier in their education programme, especially in nursing homes, they recognized these simulation situations and found no particular learning outcomes in them. It may also indicate that dementia is of little interest to nursing students, despite the fact that according to ICD-10 [44], dementia disorders constitute a mental illness. Alternatively, it can be understood as a desire for new experiences. New situations that they have not encountered before are in all likelihood more challenging than a situation with which they are more familiar.

It seems important for each student to try to play the nurse role. The statements show that the students prepared well for the patient situation when they were playing the role of the nurse, while they were less prepared for the other situations. To ensure that students prepare to face all situations, the distribution of roles can be done when students attend the Simulation Department. In this course, the students had the opportunity to distribute the roles within the group in advance. In summary, the results of the present study seem to indicate that encountering difficult situations in a safe simulation environment increases students’ competence to become aware of their own affective pattern and responses.

Blended learning often consists of a combination of classroom lectures and other digital and non-digital methods [26]. This programme replaced classroom lectures by video based asynchronous lectures. Research shows that both students and healthcare workers appreciate asynchrony, digital teaching and lectures [45]. A challenge for educators is to meet 21st^.^ century students’ expectations of flexible, digital teaching [46]. Although students’ digital competence is described in Norwegian education ministry documents [47], there is still no corresponding qualifications required for university lecturers, despite the fact that students call for more flexible teaching methods [48].

The present study revealed that the role play in the Simulation Department was important for learning outcomes. This implies that the active learning part of blended learning should be highly valued and increased. The higher the students’ activity level the greater is their learning potential [49]. Students did not participate in the planning of the course. More involvement from students in the early phase might have revealed some alternatives and logistical solutions. Perhaps active student involvement should start even earlier with an iterative process to identify what students need and involving them in the development of learning material based on their requirements. A Norwegian study has identified five important learning student learning needs during the development of new technological learning material: clarification of learning expectations, help to recognize the bigger picture, stimulation of interaction, creation of structure and context specific content [50].

This study contributes information about learning methods to enhance communication skills among nursing students preparing for mental health nursing. The results show that students in large classes with limited resources assess the blended learning method as leading to the achievement of the desired learning outcome to a high degree. Blended learning makes it possible to design courses that are more flexible than those based on ordinary lectures in auditoriums. This method is also transferable to other topics and courses.

### Methodological considerations

A strength of this study is that all students who participated had the opportunity to state their opinions of the course, not only a selected group. One hundred students answered the questionnaire, which ensures anonymity.

The main methodological limitation is the instrument for data collection. Teachers and researchers at the University developed the questionnaire based on the learning outcomes for the specific course. The questionnaire was not pre-tested or validated with other instruments.

Some of the participants filled out the questionnaire a few weeks after the course. This might represent another limitation as the passage of time can affect the memory and therefore influence the accuracy of the answers. The time required to complete the questionnaire may also have reduced the number of responses (59.2%).

The qualitative part of the study may have some limitations. Only a small number of students chose to answer the open-ended questions. Many used keywords, making it difficult to categorize the responses due to the risk of losing their full meaning. Furthermore, conducting research on one’s own students always involves an ethical challenge for the researcher [51]. This challenge is primarily related to qualitative studies where the researcher (teacher) meets the respondent (student). In the present study, the respondents are totally anonymous despite the open-ended questions. In addition, the researcher was not present when the students completed the questionnaire, thus had no possibility to influence the answers.

Evaluating one’s own courses could involve a risk of wanting the results to be better than they actually are. To ensure analytical distance, two of the authors (M.F and S.H) analysed the open-ended responses together [52].

## Conclusion

Blended learning is a relevant method in communication courses, especially the active learning part comprising role play in a Simulation Department. The present findings demonstrate that students learn more by practicing the nurse role than being an observer. The reason for this is that they were prepared for their own performance. Videotaping the role play provides a unique opportunity to reflect on one’s own performance. However, the students rated this method somewhat lower than role play alone. Our findings indicate that the method requires a cautious and carefully planned approach with sufficient time for each student to be seen and given feedback.

It is difficult to ignore the finding that feedback from an experienced teacher or standardized patient is important for the perceived learning outcome. The students themselves recommended the use of teachers in all role play. There might be two alternative strategies for achieving a higher perceived learning outcome: 1. Transferring resources from the reflection group to allow time for standardized, immediate feedback. 2. More time for the reflection group so that all video scenarios can be seen and discussed, thus fulfilling the students’ need to be seen individually.

Early student involvement and participation in developing the learning material is recommended, as it might increase the quality of the digital resources by tailoring them to the needs of the students.

## Additional file

Additional file 1: Questionnaire. English version. (DOCX 21 kb)

## Abbreviations

EQF: European qualification framework
ICD-10: International statistical classification of diseases and related health problems
INN University: Inland Norway University for applied sciences
JCAHO: The joint commission on accreditation of healthcare organizations
LMS: Learning management system
NSD: Norwegian centre for research data
SPSS: Statistical package for the social sciences
