# Supplementary material for: Communication in mental health nursing - Bachelor Students' appraisal of a blended learning training programme - an exploratory study
Source: BMC Nurs. 2018 May 15;17:20. doi: 10.1186/s12912-018-0288-9 (PMC5952371; doi:10.1186/s12912-018-0288-9)
Supplement: Supplementary file 1 — Questionnaire. English version. (DOCX 21 kb) [file 12912_2018_288_MOESM1_ESM.docx]

# Students’ assessment of the use of role-play and video recording in training communication skills

## Background information

**Campus (please mark with cross)**

| K (small campus) |  |
| --- | --- |
| E (large campus) |  |

**Gender (please mark with cross):**

| Male |  |
| --- | --- |
| Female |  |

**Age-bins (please mark with cross):**

| >20 |  |
| --- | --- |
| 21-29 years |  |
| 30 > |  |

**Native language (please mark with a cross)**

| Norwegian |  |
| --- | --- |
| Foreign |  |

**Health care work experience before starting nursing education (please mark with cross)**

| Yes |  |
| --- | --- |
| No |  |

## Usefulness of role-play and video recording in training communication skills

Below you will find two statements. Please circle the number that best agrees with your opinion.

**Statement1:**

I learn more about how I can communicate by using role-play than I would without using role-play. Totally disagree - totally agree.

1 2 3 4 5

Totally disagree totally agree

**Statement 2:**

I learnt more about my own communication skills through seeing myself on film than I would have done without using film.

1 2 3 4 5

Totally disagree totally agree

## Assessment of own learning outcomes

Below you will find some of the learning goals that were set for the teaching and exercise programme. The numbers indicate to what extent you think you have achieved the goals (learning outcomes) on a scale from 1 to 5, where 1 = "Not at all" and 5 = "To a high agree". Please circle the number that you feel is right for you.

**I am familiar with the key terms in Assisting Communication.**

1 2 3 4 5

Not at all to a high degree

**I am familiar with the various elements in “Active listening”**

1 2 3 4 5

Not at all to a high degree

**I am familiar with and have an understanding of how important it is for the nurse to be emotionally aware and emotionally tolerant when meeting patients and relatives.**

1 2 3 4 5

Not at all to a high degree

**I can combine theoretical and practical knowledge when meeting patients and relatives suffering from psychological problems.**

1 2 3 4 5

Not at all to a high degree

**I reflect on my own communication methods when meeting patients, relatives and fellow students/colleagues.**

1 2 3 4 5

Not at all to a high degree

## Importance of the methods used in the teaching and practice programme.

Please specify how you assess the importance of the methods listed below to achieve the goals of Communication Programme. Circle the number that best fits your assessment. 1 = «Not at all», 5 = « to a high degree ».

**Importance of the materials put out on Fronter.**

1 2 3 4 5

Not at all to a high degree

**Importance of training through role-play**

1 2 3 4 5

Not at all to a high degree

**Importance of training through role-play combined with film**

1 2 3 4 5

Not at all to a high degree

**Importance of training through role-play, film and reflection on film**

1 2 3 4 5

Not at all to a high degree

## Assessment of the patient cases

We ask you to assess which of the patient cases had the best learning outcome for you. Please, circle the answer that you feel is right for you:

**Anxiety/depression Dementia Psychosis Relatives**

**Please, can you describe your reason briefly?**

|  |
| --- |

## Please, can you describe which part of the programme provided the best learning outcome?

|  |
| --- |

## If you have any suggestions or recommendations for future communication courses, please note them here.

|  |
| --- |

**Thank you for your answers.**
